# Baby Food Pouches, Baby-Led Weaning, and Iron Status in New Zealand Infants: An Observational Study

**DOI:** 10.3390/nu16101494

**Published:** 2024-05-15

**Authors:** Neve H. McLean, Jillian J. Haszard, Lisa Daniels, Rachael W. Taylor, Benjamin J. Wheeler, Cathryn A. Conlon, Kathryn L. Beck, Pamela R. von Hurst, Lisa A. Te Morenga, Jenny McArthur, Rebecca Paul, Ioanna Katiforis, Kimberley J. Brown, Madeline C. Gash, Madeleine M. Rowan, Maria Casale, Alice M. Cox, Emily A. Jones, Rosario M. Jupiterwala, Bailey Bruckner, Liz Fleming, Anne-Louise M. Heath

**Affiliations:** 1 Department of Human Nutrition, University of Otago, Dunedin 9016, New Zealand; neve.mclean@otago.ac.nz (N.H.M.); ioanna.katiforis@postgrad.otago.ac.nz (I.K.); madelinegash9@gmail.com (M.C.G.); maddie.rowan@otago.ac.nz (M.M.R.); bailey.bruckner@outlook.com (B.B.); liz.fleming@otago.ac.nz (L.F.); 2 Biostatistics Centre, University of Otago, Dunedin 9016, New Zealand; jill.haszard@otago.ac.nz; 3 Department of Medicine, University of Otago, Dunedin 9016, New Zealand; lisa.daniels@otago.ac.nz (L.D.); rachael.taylor@otago.ac.nz (R.W.T.); jenrose19@hotmail.com (J.M.); coxal603@student.otago.ac.nz (A.M.C.); 4 Department of Women’s and Children’s Health, University of Otago, Dunedin 9016, New Zealand; ben.wheeler@otago.ac.nz; 5 School of Sport, Exercise and Nutrition, Massey University, Auckland 0632, New Zealand; c.conlon@massey.ac.nz (C.A.C.); k.l.beck@massey.ac.nz (K.L.B.); p.r.vonhurst@massey.ac.nz (P.R.v.H.); r.paul@massey.ac.nz (R.P.); dietitian.kimberley@outlook.com (K.J.B.); m.casale@massey.ac.nz (M.C.); e.jones@massey.ac.nz (E.A.J.); r.p.monzales@massey.ac.nz (R.M.J.); 6 Research Centre for Hauora and Health, Massey University, Wellington 6140, New Zealand; l.temorenga@massey.ac.nz

**Keywords:** infants, iron status, complementary feeding, baby food pouches, baby-led weaning, New Zealand

## Abstract

Iron deficiency in infants can impact development, and there are concerns that the use of baby food pouches and baby-led weaning may impair iron status. First Foods New Zealand (FFNZ) was an observational study of 625 New Zealand infants aged 6.9 to 10.1 months. Feeding methods were defined based on parental reports of infant feeding at “around 6 months of age”: “frequent” baby food pouch use (five+ times per week) and “full baby-led weaning” (the infant primarily self-feeds). Iron status was assessed using a venepuncture blood sample. The estimated prevalence of suboptimal iron status was 23%, but neither feeding method significantly predicted body iron concentrations nor the odds of iron sufficiency after controlling for potential confounding factors including infant formula intake. Adjusted ORs for iron sufficiency were 1.50 (95% CI: 0.67–3.39) for frequent pouch users compared to non-pouch users and 0.91 (95% CI: 0.45–1.87) for baby-led weaning compared to traditional spoon-feeding. Contrary to concerns, there was no evidence that baby food pouch use or baby-led weaning, as currently practiced in New Zealand, were associated with poorer iron status in this age group. However, notable levels of suboptimal iron status, regardless of the feeding method, emphasise the ongoing need for paying attention to infant iron nutrition.

## 1. Introduction

The evolution of infant nutrition practices in many countries has seen a rise in the popularity of innovative complementary feeding methods such as baby food pouches and baby-led weaning [1,2,3,4,5,6]. This shift in infant feeding approaches has raised concerns about their potential impact on infant nutrition and health [3,7,8,9,10]. One particular concern is regarding the potential risk of iron deficiency. Iron deficiency is a significant concern due to the low iron content of baby food in commercial baby food pouches [5] and the limited selection of iron-rich foods appropriate for self-feeding in baby-led weaning [3,7]. A compromised iron status has immediate and potentially long-term developmental consequences [11,12]. Therefore, understanding the associations between these emerging complementary feeding practices and iron status is crucial.

Despite the growing popularity of baby food pouches and baby-led weaning, a notable gap exists in the literature regarding their potential influence on infant iron status. To date, no studies have explored whether the use of baby food pouches impacts infants’ iron status. Similarly, while several investigations suggest a lower iron intake with baby-led weaning than with traditional spoon-feeding [13,14,15], the association between baby-led weaning and iron status itself has not been determined. While randomised controlled trials have found no evidence that a *modified* version of baby-led weaning increased the risk of iron deficiency at 12 months of age [16,17], these interventions provided advice and support specifically encouraging the introduction of iron-rich foods, making it unclear whether baby-led weaning practices as followed in the community, without such interventions, affect iron status.

Assessing the relationship between early feeding practices and iron status is important because iron status is of particular concern during late infancy. Existing studies in New Zealand have reported prevalence rates of iron deficiency (encompassing early “functional” iron deficiency and iron deficiency anaemia) ranging from 11 to 14% and iron deficiency anaemia ranging from 5 to 7% [16,18,19]. However, these estimates are based on data collected more than two decades ago [18,19] or from small, non-representative samples [16] and predate the widespread use of baby-led weaning and baby food pouches for introducing complementary foods.

This study aims to evaluate the relationship between the use of baby food pouches and baby-led weaning at “around” 6 months of age (the recommended age for introducing complementary foods in New Zealand [20]) and the subsequent iron status (determined using body iron and haemoglobin concentrations) in New Zealand infants 7–10 months of age. The objectives were to (1) estimate iron status in New Zealand infants 7–10 months of age, (2) determine whether feeding approach (commercial baby food pouch use or baby-led weaning) at “around” 6 months predicts the current iron status, and (3) explore associations between other early feeding practices and the current iron status.

## 2. Materials and Methods

### 2.1. Study Design 

Detailed methods have been described elsewhere [21], so only information relevant to this study is included here. First Foods New Zealand (FFNZ) was an observational study of nutrition and health, including the use of baby food pouches and baby-led weaning, in infants aged 6.9 to 10.1 months in New Zealand. All adult participants provided written informed consent. This study had ethical approval from the Health and Disability Ethics Committees New Zealand (19/STH/151) and was registered with the Australian New Zealand Clinical Trials Registry (ACTRN12620000459921).

A sample size of 625 was estimated to provide 80% power (α = 0.05) to detect a 5.0 μg/L mean difference in plasma ferritin concentrations (SD = 16.8 μg/L) in infants following baby-led weaning compared to those following traditional spoon-feeding, which was a primary objective of the FFNZ study [21]. This calculation assumed 70% complete biochemical data [16] and a prevalence of 28.3% for baby-led weaning [4]. Sample size calculations for pouch use were not possible because of the absence of prevalence data in New Zealand or internationally.

### 2.2. Participant Eligibility and Recruitment

Participants were infant–main carer pairs. The study’s target age range of 7 to 10 months was defined as 30 to 44 weeks of age (approximately equivalent to 6.9 to 10.1 months). Infant participants had to be within this age range, live in either Dunedin or Auckland (large urban centres in the South and North Islands of New Zealand), and must not have participated in a nutrition intervention study in which the parent was asked to change how their infant was fed. Adult participants were 16 years of age or older and could communicate in English. Most adult participants (99%) were parents. Therefore, the term “parents” is used to refer to adult participants from this point onwards. Participants were recruited through advertisements or word of mouth between July 2020 and February 2022. The study aimed to minimise recruitment bias, targeting all infants rather than specifically those who used baby food pouches or baby-led weaning *per se* and a diverse range of ethnic and socioeconomic groups. Participants received a voucher valued up to NZD 150 to acknowledge their time and expenses. 

### 2.3. Procedure

All information related to the current analyses was obtained during three main study visits that occurred over two weeks following enrolment. The first main visit, held in the participant’s home, involved collecting written informed consent, a 24 h diet recall, infant anthropometric measurements, and a questionnaire that included demographic and infant feeding measures. The second main visit, held either in study clinic rooms or in the participant’s home, involved collecting a second 24 h diet recall. During the third main visit, conducted in study clinic rooms (Dunedin) or a local blood testing facility (Labtests, Auckland), a blood sample was collected from the infant and analysed to assess their iron status. 

### 2.4. Questionnaire Data 

An online questionnaire was administered that gathered demographic and infant feeding measures. The demographic information collected included infant and parent age, infant sex, and infant ethnicity (classified by New Zealand Census categories [22]). Participants who reported identifying as belonging to more than one ethnic group were assigned to a single group, prioritised in the following order (from highest to lowest): Māori, Pacific, Asian, Others, and European [22]. Information was collected about whether the infant was born term or preterm (i.e., less than 37 weeks gestation), parental employment status, highest educational level, maternal parity, self-reported maternal height and weight, household composition, and use of early childhood education or childcare. Additionally, area-level socioeconomic deprivation, as a proxy for socioeconomic status, was determined using the New Zealand Index of Deprivation (NZDep) [23] based on home addresses. Infant feeding measures included the age when solid foods were introduced (with monthly response option categories), the use of baby food pouches at “around 6 months”, the use of baby-led weaning at “around 6 months”, the duration of exclusive breastfeeding (with monthly response option categories), the current breastfeeding status, the consumption of iron-fortified infant cereal or red meat “around 6 months” of age, and the use of supplements (in the past month). The average daily nutrient intake from supplements was determined by multiplying the nutrient content per unit (obtained from the product label or website) by the estimated frequency of supplement consumption in the past month. Infants who had consumed a supplement containing iron in the past month were classified as current iron supplement users. 

The questionnaire data were used to define infant feeding approaches based on feeding practices at “around 6 months of age”. The focus on the relationship between feeding practices at around 6 months of age, rather than at the time of the blood sample, and the subsequent evaluation of iron status at 6.9–10.1 months was to allow time for differences in diet to manifest as measurable differences in iron status. Three categories of baby food pouch use were defined: “Frequent pouch user” (given food from a pouch at least 5 times per week at around 6 months); “Non-frequent pouch user” (given food from a pouch at least once at around 6 months but fewer than 5 times per week); and “Non-pouch user” (not been given food from a pouch at around 6 months of age or had never been given food from a pouch). The study focused on the use of commercial ‘ready-to-eat’ baby food pouches. Similarly, three categories were established for baby-led weaning practices: “Full baby-led weaning” (always or mostly the baby feeding themselves at around 6 months); “Partial baby-led weaning” (about half spoon-fed by an adult and half the baby feeding themselves at around 6 months); and “Traditional spoon-feeding” (always or mostly spoon-fed by an adult at around 6 months) [4]. The “other early feeding practices” of interest were exclusive breastfeeding up to around 6 months of age, the age of introduction to solid foods, and iron-fortified infant cereal or red meat consumption at around 6 months of age.

### 2.5. Anthropometric Assessment

Trained researchers measured infants’ weight and length using calibrated equipment and standardised techniques [24]. Two measurements were taken, with a third taken if the difference between duplicate measures exceeded the specified threshold [24]. Infant weight was measured using an electronic scale (models 334 and 354; Seca), and length was measured using a 99 cm length board (model SE210; Seca). Self-reported adult weight and height were assessed via a questionnaire. Infant and adult BMIs were calculated (weight in kg divided by length or height in meters squared), and infant BMI z-scores were determined using the World Health Organisation Child Growth Standards [25,26].

### 2.6. Assessment of Infant Formula Intake 

Infant formula intake data, necessary for subsequent statistical analyses (described below), were obtained from two 24 h diet recalls conducted by trained interviewers. Most of the first interviews took place in participants’ homes, and the second took place in either homes or study clinic rooms. The 24 h diet recalls used a multiple-pass method, following the four stages used in the 2008/09 New Zealand Adult Nutrition Survey [27], to capture the infant’s food and beverage consumption from midnight to midnight on the previous day. For cases involving caregivers other than the parent (e.g., childcare workers, grandparents), a food diary was provided and used by the other caregiver to document the child’s consumption on the day being recalled. Rigorous quality control procedures were implemented to ensure data integrity.

To account for within-person variance in intake, a second 24 h diet recall was conducted. The participant’s two 24 h diet recalls took place on different days of the week to capture the variation in intake between days, including weekend days, with all days of the week represented by the sample of participants as a whole.

Trained research staff entered 24 h diet recall data into FoodWorks 10 (Xyris Software, Australia) following a standardised protocol. As FOODfiles 2018, the food composition database for New Zealand [28], lacked commercial infant food and infant formula data, the research team added information for commercial baby food products and infant formulas on the market at the time of the study [5]. The added data comprised 385 baby food products and 104 infant formulas.

### 2.7. Blood Collection and Laboratory Methods

During the third main visit, a non-fasting venepuncture blood sample (3 mL EDTA anticoagulated vacutainer blood collection tube; Becton Dickinson and Company, Auckland, New Zealand) was collected from infants in study clinic rooms (Dunedin) or a local blood testing facility (Labtests, Auckland, New Zealand). Participants were advised to reschedule their appointment if their child was unwell. Before the visit, participants were given a tube of Ametop gel (Smith & Nephew, Auckland, New Zealand) local anaesthetic and Opsite Flexigrid (Smith & Nephew, Auckland, New Zealand) dressing, with a video and written instructions on how to apply it.

Blood samples were analysed for haemoglobin concentration (from a complete blood count (CBC)) with an automated haematology analyser (Sysmex XN20, Sysmex Corporation, Kobe, Japan) and plasma ferritin by a sandwich assay using an E602-Cobas 8000 (Roche Diagnostics, Indianapolis, IN, USA). Blood samples collected in Dunedin were analysed for whole blood CBC and plasma ferritin on the day of collection at Southern Community Laboratories. Blood samples collected in Auckland were analysed on the day of blood collection for whole blood CBC at Labtests, with the remaining sample transported in chilled storage to Southern Community Laboratories in Dunedin. Chilled transported samples were centrifuged and then assayed for ferritin by Southern Community Laboratories within seven days of collection. Both laboratories are clinical laboratories that regularly conduct internal and external quality control procedures. 

Biochemical results that fell outside predefined clinical reference ranges for CBC indices or plasma ferritin were reviewed by a paediatrician (BJW) who proposed a likely explanation of the results to the infant’s general practitioner so that they had support for interpreting the “out of range” measurement in these apparently healthy infants. The participant was then promptly contacted, informed about the “out of range” finding, and advised to seek guidance from their general practitioner for further evaluation and appropriate medical advice if indicated.

Aliquots of the remaining plasma were stored at the Department of Human Nutrition (the University of Otago, New Zealand) at −70 °C until subsequent analysis. Plasma concentrations of soluble transferrin receptor (sTfR), C-reactive protein (CRP), and α-1-acid glycoprotein (AGP) were measured using a Hitachi Cobas C311 automatic electronic analyser (Roche Diagnostics) at the Department of Human Nutrition, the University of Otago. The accuracy and precision of analyses were checked using controls, as well as in-house pooled samples (every 20 samples). The analysed mean (SD, CV) values for the multilevel sTfR (Roche Diagnostics, Indianapolis, IN, USA) assay were as follows: 2.21 mg/L (0.05 mg/L, 2.4%) and 7.23 mg/L (0.08 mg/L, 1.1%) compared to the manufacturer’s concentrations of 2.31 mg/L and 7.47 mg/L, respectively; for the CRP (Roche Diagnostics, US) assay, 10.7 g/L (0.3 g/L, 3.0%) compared to the manufacturer’s concentration of 10.7 g/L; and for the multilevel AGP (Roche Diagnostics, Indianapolis, IN, USA) assay, 0.48 mg/L (0.01 mg/L, 2.0%) and 0.82 mg/L (0.01 mg/L, 1.2%) compared to the manufacturer’s concentrations of 0.50 mg/L and 0.85 mg/L, respectively. 

### 2.8. Statistical Analysis

Stata statistical software version 17.0 [29] was used for all statistical analyses. Prior to the analysis, plasma ferritin and sTfR concentrations were adjusted to account for inflammation using the BRINDA (Biomarkers Reflecting Inflammation and Nutritional Determinants of Anaemia) statistical method [30,31]. Briefly, this method involves the use of regression models to estimate the expected iron biomarker concentration in the absence of inflammation, based on the observed CRP and AGP concentrations. After adjusting for inflammation, plasma ferritin and sTfR concentrations were used to calculate body iron, as described in Cogswell et al. [32]. First, the sTfR concentrations determined using the Roche assay were converted to values equivalent to those of the Flowers assay using the following equation: 1.5 × Roche sTfR + 0.35 mg/L [32]. Then, body iron was calculated using the following equation by Cook et al. [33]:

$$Body\;iron\;\left(mg/kg\right)=-\frac{\left[log10\left(\frac{sTfR\times 1000}{ferritin}\right)-2.8299\right]}{0.1207}$$

Simple descriptive statistics were calculated to describe the characteristics of infants and parents in the whole FFNZ sample and in those with iron status data and to describe measures of iron status and inflammation in those with iron status data. Iron status categories were defined using plasma ferritin concentrations, body iron concentrations, and haemoglobin concentrations. The iron status categories are defined in Table 1. 

The proportion of infants in each of the iron status categories was reported for the study sample and weighted by ethnicity and household socioeconomic deprivation to better reflect the New Zealand population [36], generating weighted estimates and 95% CIs for the prevalence. 

Plasma ferritin concentration, body iron concentration, and iron sufficiency were examined in relation to use of baby food pouches, baby-led weaning, and the other early feeding practices. Due to the small group sizes for the iron status categories, only relationships with iron sufficiency could be examined. Linear regression was used to estimate the associations with plasma ferritin and body iron concentrations, and logistic regression was used to estimate associations with iron sufficiency. Because distributions of plasma ferritin concentrations were positively skewed, these were log-transformed prior to analysis, geometric means were reported, and estimates from the regression models were back-transformed to be the ratio of geometric means for the subgroups (reported as percent differences). Both unadjusted and adjusted models were presented. It was decided a priori to include the demographic variables such as infant age, infant sex, and household socioeconomic deprivation in the adjusted models. Additionally, due to their potential rapid and marked impact on iron status, current infant formula intake and iron supplement use were also included *a priori* in the adjusted models. Residuals of the linear models were plotted and visually assessed for homoscedasticity and normality.

Exploratory analyses of demographic and current dietary characteristics that could be predictors of body iron concentrations and iron sufficiency are presented in Table S1 as context for the main analyses described above. Descriptive statistics were used to describe body iron and iron sufficiency proportions by subgroup. Differences in mean body iron concentrations and the proportions of iron sufficiency between the subgroups were assessed using one-way ANOVA F tests and chi-squared tests (or Fisher’s exact test when a subgroup contained *n* < 10), respectively. The resulting *p*-values for each test are presented, with *p* < 0.05 considered to indicate statistical significance. 

## 3. Results

### 3.1. Participant Recruitment and Inclusion

**Figure 1:**
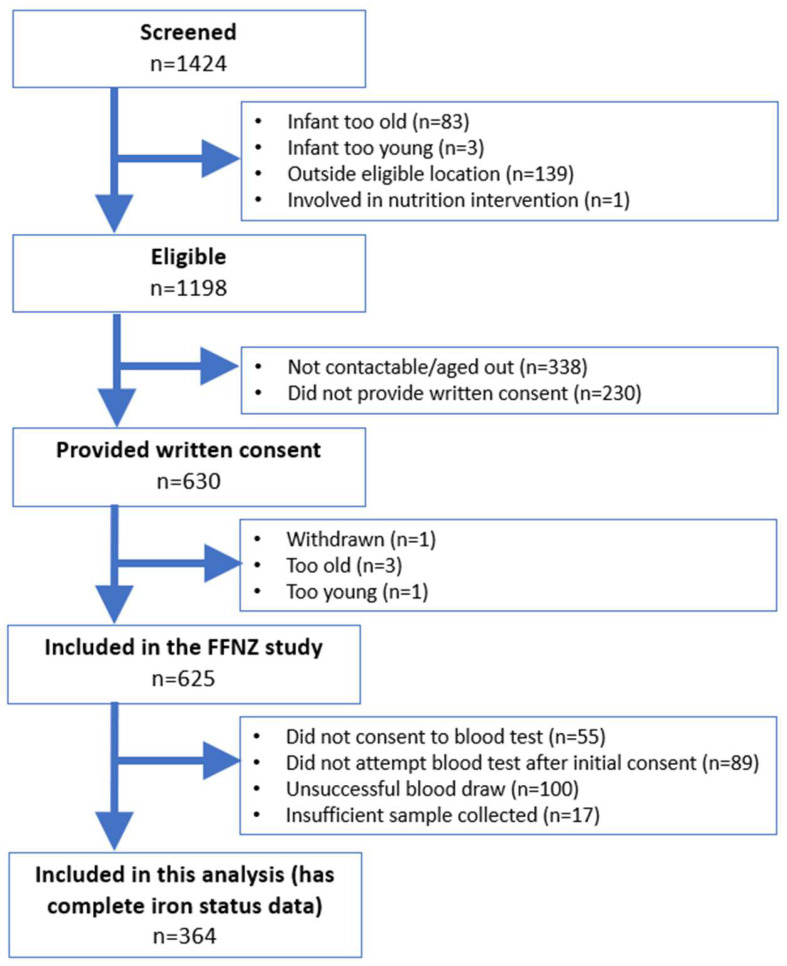
Flow diagram illustrating participant recruitment and inclusion.

Participant recruitment and inclusion criteria for the current study are outlined in Figure 1. Initially, 630 infant–parent pairs consented to participate in the FFNZ study. Of these, one withdrew from the study, and four were outside the target age range at the time of consent, resulting in 625 participants. Of these participants, a total of 364 (58%) provided complete iron status data (i.e., haemoglobin, plasma ferritin, sTfR, CRP, and AGP) and were therefore included in the current analyses. The reasons for not providing complete iron status data were not consenting to the blood test (*n* = 55), not attempting the blood test after the initial consent (*n* = 89), unsuccessful attempts at obtaining a blood sample (*n* = 100), and insufficient samples collected (*n* = 17). Unsuccessful attempts at obtaining a blood sample were because of challenges that are particularly apparent in this age group such as infant movement or difficulty accessing the vein due to the high amount of body fat covering it.

### 3.2. Participant Characteristics 

Table 2 describes the characteristics of participants in this study and in the FFNZ study as a whole. The average age of infants who provided complete iron status data was 8.3 months (range: 6.9 to 10.1 months), and the mean BMI z-score was 0.3. The majority (66%) of these infants were identified by their parents as having “New Zealand and other European” ethnicities, with 17%, 4%, and 11% identified as having “Māori”, “Pacific”, and “Asian” ethnicities, respectively. Most infants were born at term (93%), and 44% were female. Parents, predominantly the infant’s mother (99%), had a mean age of 33 years, with 67% having completed university and 65% not currently employed. Approximately half (49%) were primiparous. Infants with high socioeconomic deprivation were somewhat under-represented, comprising 24% of those participants with iron status data compared to 28% in a national dataset of New Zealand households with infants [36]. Nearly all infants (98%) had been breastfed at some point, and 68% were breastfed at the time they took part in the study (mean age 8.3 months). Almost half (47%) were fed infant formula on one or more of the 24 h diet recall days. Approximately one in three infants (37%) were first introduced to solids at 6 months of age, with the majority (97%) starting solids before 7 months of age and only 1% starting solids before 4 months of age. Fewer than 1% of infants used iron supplements. 

Participants with iron status data were similar to those in the whole sample for all characteristics shown (Table 2), with the exception of infant ethnicity. A greater proportion of infants with iron status data were identified by their parents as having “NZ and other European” ethnicities (66% compared with 55%), while a lower proportion were identified as having “Māori”, “Pacific”, and “Asian” ethnicities. 

### 3.3. Iron Status of New Zealand Infants

The sample and weighted estimates for measures of iron status and inflammation are presented in Table 3. After weighting for ethnicity and socioeconomic deprivation level, the mean body iron concentration was 3.0 mg/kg (95% CI: 2.7 to 3.4 mg/kg). The estimated prevalence of infants with suboptimal iron status was 22.9%, comprising 8.9% (95% CI: 6.3% to 12.3%) with iron depletion, 11.1% (95% CI: 8.1% to 15.1%) with early “functional” iron deficiency, and 2.9% (1.5% to 5.5%) with iron deficiency anaemia. The proportions were similar for the study sample. 

### 3.4. Baby Food Pouch Use as a Predictor of Iron Status

When compared to non-pouch users, the adjusted geometric mean plasma ferritin concentration was 16% higher (95% CI: −3 to 38) for non-frequent pouch users and 5% higher (95% CI: −16 to 31) for frequent pouch users, but neither of these differences were statistically significant (Table 4). Similarly, both frequent and non-frequent baby food pouch use at around 6 months predicted body iron concentrations and iron sufficiency within the sample; however, after adjusting for potential confounding factors (including infant formula consumption), these associations were attenuated, and statistically significant differences were observed only for non-frequent pouch users (0.75 mg/kg (95% CI: 0.02 to 1.48) higher mean body iron concentration and 2.2 (95% CI: 1.15 to 4.21) times higher odds of iron sufficiency compared to non-pouch users).

### 3.5. Baby-Led Weaning as a Predictor of Iron Status

There were no statistically significant differences in plasma ferritin concentrations by baby-led weaning use in either the adjusted or the unadjusted analyses (Table 5). When compared to infants following traditional spoon-feeding, the adjusted geometric mean plasma ferritin concentration was 14% lower (95% CI: −33 to 10) for infants who had been following partial baby-led weaning at 6 months of age and 4% higher (95% CI: −18 to 30) for infants following full baby-led weaning, but neither of these differences were statistically significant. Neither partial nor full baby-led weaning at around 6 months of age significantly predicted current body iron concentrations: the adjusted mean difference in body iron concentration was −0.34 mg/kg (95% CI: −1.37 to 0.69) for infants who had been following partial baby-led weaning and 0.02 mg/kg (95% CI: −0.92 to 0.97) for infants following full baby-led weaning when compared to those following traditional spoon-feeding. Partial, but not full, baby-led weaning at 6 months of age predicted lower odds of iron sufficiency among infants within the sample, but differences were not statistically significant when the data were adjusted for potential confounding factors: adjusted OR 0.52 (95% CI: 0.24 to 1.13) for infants following partial baby-led weaning compared to traditional spoon-feeding and 0.91 (95% CI: 0.45 to 1.87) for infants following full baby-led weaning compared to traditional spoon-feeding. 

### 3.6. Other Feeding Characteristics at 6 Months of Age and Current Iron Status 

The exploration of other early feeding characteristics, other than baby food pouch use and baby-led weaning, as predictors of body iron concentrations and iron sufficiency is shown in Table 6. Among the feeding characteristics investigated, only late introduction to solids (≥7 months) predicted measures of current iron status. Adjusted analyses showed that introduction to solids at ≥7 months was associated with current 41.8% (95% CI: 3.8 to 64.6%) lower plasma ferritin concentrations, 2.19 mg/kg (95% CI: 0.18 to 4.20 mg/kg) lower body iron concentrations, and lower odds of iron sufficiency (OR 0.21 (95% CI: 0.05 to 0.96)) when compared to infants who were introduced to solids at 6 months of age.

### 3.7. Demographic and Later Feeding Characteristics and Current Iron Status 

The study also explored demographic and later feeding characteristics as predictors of current body iron concentrations and iron sufficiency (Table S1; Supplementary Materials), providing context for the main analyses described above. Infants who were older, males, preterm births, currently breastfeeding, or currently consuming no infant formula had lower body iron levels and were less likely to be iron-sufficient (all *p* < 0.05). Those at risk of overweight or who were not in formal childcare were also less likely to be iron-sufficient (*p* < 0.05). Additionally, infants who had never been breastfed (although there were only nine of them) had significantly higher body iron levels than those who had been breastfed.

## 4. Discussion

This study presents the first data internationally on the relationship between iron status and the use of baby food pouches and baby-led weaning in infants. This assessment is important considering the recent concerns raised about the potential health implications of these feeding methods [3,7,8,9,10]. The overall prevalence of suboptimal iron status among New Zealand infants aged 6.9 to 10.1 months was estimated to be 23% (although only 3% had iron deficiency anaemia), emphasising the importance of determining whether these new feeding approaches are associated with iron status. Infants in the study who consumed baby food from pouches (as frequent or non-frequent users) at around six months of age had higher body iron concentrations and higher odds of being iron-sufficient than those who did not. However, after adjusting for several demographic and dietary factors, including infant formula intake, these associations were observed only in non-frequent pouch users. No evidence indicated that baby-led weaning was associated with either body iron concentration or iron sufficiency. 

The estimated prevalence of iron deficiency (defined as early “functional” iron deficiency or iron deficiency anaemia) in this group of 7–10 month old New Zealand infants was 14%, although only 3% had iron deficiency anaemia (the level of iron deficiency supported by more robust evidence as indicating functional consequences). Few other data on iron status exist for infants in New Zealand [16,18,19], but prevalence estimates in those studies are generally consistent with those seen here. Earlier population-based surveys in New Zealand infants reported prevalence estimates of iron deficiency ranging from 11 to 14%, with iron deficiency anaemia ranging from 6 to 7% [18,19]. Similarly, more recent data from a randomised controlled trial in New Zealand indicated that at 12 months of age, 12% of infants in the control group had iron deficiency, while 5% had iron deficiency anaemia [16]. The prevalence estimates in the current study are also comparable to those seen for toddlers in the United States, where a prevalence of iron deficiency of 15% was found between 2003 and 2010 using NHANES data [39]. 

Although the use of baby food pouches appears to be growing in popularity, their potential impact on infant nutrition and health is poorly understood. Contrary to concerns that baby food pouch use could negatively affect infants’ iron status [5], the current findings suggest that infants who frequently consumed baby food from pouches at around 6 months of age had significantly higher body iron concentrations and were over two times more likely to be iron-sufficient than non-users at the time of assessment (one to four months later). However, controlling for potential confounders substantially attenuated most of these associations which suggested that other factors, such as infant formula intake, played an important role in the observed association between baby food pouch use and iron status. In *post hoc* analyses, infants who were frequently pouch-fed were significantly more likely to consume infant formula (68% compared to 27% among non-pouch users). Infant formula in New Zealand has high concentrations of iron, mandated to be 0.2–0.5 mg/100 kJ (equivalent to approximately 0.6–1.4 mg/100 mL of prepared product) [40], reflecting the need for high levels of fortification due to the reduced bioavailability of iron in infant formula. It is important to note that the message here is not to discourage breastfeeding, which has many benefits for both the mother and the infant [41]. Instead, it highlights the importance of ensuring that breastfed infants receive adequate amounts of iron through other dietary sources during the complementary feeding period [42]. 

Moreover, although non-frequent pouch use was still a positive predictor of body iron concentrations and being iron-sufficient even after controlling for several demographic and dietary factors, establishing a *true causal* positive association with non-frequent pouch use is challenging. Most importantly, baby food in pouches has a low iron content [5]. Furthermore, non-frequent baby food pouch users tend to differ from those who do not use baby food pouches in various ways, such as introducing solids earlier and being more likely to use formal childcare than non-users [43]. Exploratory analyses showed that both these practices were positively associated with measures of iron status (see Table S1; Supplementary Materials). The observed positive associations between non-frequent pouch use and iron status outcomes in the current study are likely confounded by other variables such as these, particularly as the same association is not seen amongst those who use pouches more frequently. Ultimately, although it is unlikely that consuming baby food from pouches in itself enhances iron status at this age, it is also the case that this study provides no evidence that using baby food pouches is associated with poorer iron status, as suggested elsewhere [5]. 

Some health professionals have expressed concerns that baby-led weaning may increase the risk of developing iron deficiency [3,7]. In addition, in New Zealand, infant feeding guidelines do not endorse the use of baby-led weaning as an appropriate alternative approach to infant feeding, citing a lack of conclusive evidence about its safety [44]. The present analysis provides a substantial contribution to the evidence base regarding the safety of baby-led weaning in terms of iron status. There was no evidence that full baby-led weaning was associated with lower plasma ferritin or body iron concentrations, or a lower likelihood of iron sufficiency, when compared to traditional spoon-feeding, in either the adjusted or unadjusted analyses. These findings are consistent with previous data from two randomised controlled trials that reported no evidence that *modified* baby-led weaning increases the risk for iron deficiency at 12 months of age [16,17]. However, these interventions provided advice and support to promote high-iron foods, so the current data provide the first evaluation internationally of iron status in infants who used a baby-led weaning approach in the community without any intervention. These data suggest that infants who were mostly feeding themselves at around 6 months of age were no more likely to have poor iron status than those using traditional spoon-feeding. 

Among the other early feeding practices that occur alongside baby food pouch use and baby-led weaning and were investigated in this study, age of introduction to solids was a strong predictor of current iron status. After adjustment, infants who were introduced to solids at ≥7 months had significantly lower plasma ferritin and body iron concentrations and were much less likely to be iron-sufficient at their current age than those who were introduced to solids at 6 months of age. These findings highlight the importance of promptly introducing iron-rich complementary foods around the age of 6 months, considering that iron stores are normally depleted by this age [35]. Interestingly, no association was observed between the consumption of iron-fortified infant cereals, or of red meat, *at around 6 months of age* and iron status at 7 to 10 months of age. These findings contrast with several studies [45,46,47] that have reported a positive correlation between these dietary factors and iron stores in young children. However, in the current study, the broad definition of “consumption”, encompassing retrospective reports of any red meat or iron-fortified infant cereal consumption at around 6 months, limited the ability to assess whether higher consumption levels may be associated with better iron status. Furthermore, the retrospective nature of the assessment, relying on parental recall one to four months later, may have introduced measurement errors, potentially reducing the ability to detect true differences.

This study has several strengths including that it is the first to investigate the relationship between the frequent use of baby food pouches and iron status, as well as the first to assess iron status in infants following baby-led weaning in a community setting. These findings add to the understanding of the safety of these feeding practices in infants and can inform public health recommendations on their use. Another strength is that the use of the BRINDA method [31] allowed us to address confounding by inflammation, improving the accuracy of the assessment of iron status. 

However, the current findings should be interpreted in light of a number of limitations. First, only 58% of infants provided complete iron status data, falling short of the 70% target in the FFNZ study, which led to a reduction in the statistical power and precision of the estimates. However, confidence intervals were reported to provide insights into the range of plausible values in the population. Second, infants who provided complete iron status data had slightly different ethnic distributions and were somewhat less likely to come from socioeconomically deprived areas compared to the general population. To minimise these potential biases, the estimates were weighted to match the population distributions for ethnicity and household deprivation. Third, while bone marrow examination, continuous phlebotomy, and response to iron treatment are considered gold standards for diagnosing iron deficiency, these methods were not feasible to use in this study of infants. Instead, the current study relied on biochemical markers for defining iron deficiency, which are known to be challenging to interpret in infants as thresholds for defining iron deficiency are not well established. In addition, the suitability of body iron as a measure of iron status in infants has not been validated, although it has been used in other studies of very young children [32,39]. Lastly, while the current study suggested no impact on iron status from frequent baby food pouch use or baby-led weaning at 6 months, the possibility of later effects from ongoing use cannot be ruled out. However, with respect to baby-led weaning, the Healthy Eating Guidelines for New Zealand Babies and Toddlers [20] recommend the introduction of finger foods by 8 months, so baby-led-weaning-type behaviours would be expected to increase from that age in the population in general. This suggests that differences in iron status are unlikely to appear as a result of baby-led weaning after the age investigated here (mean age 8.3 months).

## 5. Conclusions

In conclusion, this study provides the first estimates internationally of iron status in infants in relation to baby food pouch use. It also reports the first estimates of iron status in infants using a baby-led weaning approach in the community, without intervention. Our findings do not support concerns that either of these feeding practices, as they are currently practiced in New Zealand, are associated with poorer iron status in infants 7–10 months of age. However, it is noteworthy that approximately one in four infants in New Zealand had evidence of suboptimal iron status, which highlights the need for continued attention from clinicians and policy makers to ensure that infants meet their iron requirements during the complementary feeding period, regardless of the feeding method.

## Figures and Tables

**Table 1:** Criteria used to determine iron status categories.

Iron Status Category	Criteria Used
Iron-sufficient ^a^	Plasma ferritin ≥ 15 μg/L ^b^, in the absence of early “functional” iron deficiency, iron deficiency anaemia, and iron depletion
Iron-depleted	Plasma ferritin < 15 μg/L ^b^, in the absence of early “functional” iron deficiency and iron deficiency anaemia
Early “functional” iron deficiency	Body iron < 0 mg/kg ^c^ and haemoglobin ≥ 105 g/L ^d^
Iron deficiency anaemia	Body iron < 0 mg/kg ^c^ and haemoglobin < 105 g/L ^d^

^a^ Those who were determined to have other anaemia (i.e., body iron ≥ 0 mg/kg and haemoglobin < 105 g/L) were also considered iron-sufficient. ^b^ Cutoff value from Southern Community Laboratories Ltd. [34]. ^c^ Cutoff value from Cook et al. [33]. ^d^ Cutoff value from Domellöf et al. [35].

**Table 2:** Characteristics of the whole sample and those with iron status data available.

	Whole Sample (*n* = 625)	Sample with Iron Status Data ^a^ (*n* = 364)
	*n* (%), Unless Otherwise Stated	
Infant characteristics		
Age (months), mean (SD)	8.4 (0.8)	8.3 (0.8)
Female sex ^b^,	289 (46.2)	161 (44.2)
Ethnicity ^c^		
Māori	131 (21.0)	62 (17.0)
Pacific	44 (7.0)	14 (3.9)
Asian	90 (14.4)	39 (10.7)
Others	16 (2.6)	10 (2.8)
NZ and other European	344 (55.0)	239 (65.7)
Born at term ^d^	578 (92.6)	340 (93.4)
BMI z-score ^e^, mean (SD)	0.30 (1.0)	0.31 (1.0)
Respondent characteristics		
Mother	617 (98.7)	359 (98.6)
Age (years), mean (SD) ^f^	32.7 (4.9)	33.0 (4.7)
Highest level of education completed		
School (primary or secondary)	94 (15.1)	49 (13.5)
Polytechnic or similar tertiary institution	125 (20.0)	71 (19.6)
University	405 (64.9)	243 (66.9)
Maternal primiparous	303 (48.6)	178 (48.9)
Current employment status		
Employed full time	70 (11.2)	45 (12.4)
Employed part time	137 (21.9)	84 (23.1)
Other ^g^	418 (66.9)	235 (64.6)
BMI (kg/m^2^), mean (SD) ^h^	27.8 (6.4)	27.8 (6.1)
Overweight or obese, *n* (%) ^h^	348 (58.4)	217 (61.7)
Childcare use ^i^	109 (17.4)	66 (18.1)
Household factors		
Number of children living in household		
One	283 (45.4)	165 (45.5)
Two	200 (32.1)	124 (34.2)
Three or more	141 (22.6)	74 (20.4)
Number of adults living in household		
One	25 (4.0)	16 (4.4)
Two	517 (82.7)	311 (85.4)
Three or more	83 (13.3)	37 (10.2)
Household deprivation decile ^j^		
1–3 (low)	180 (28.8)	111 (30.5)
4–7	282 (45.1)	165 (45.3)
8–10 (high)	163 (26.1)	88 (24.2)
Infant feeding characteristics		
Ever breastfed	612 (97.9)	355 (97.5)
Currently breastfed	414 (66.2)	249 (68.4)
Current infant formula intake ^k^		
No formula	319 (51.0)	191 (52.5)
<1000 kJ/d	73 (11.7)	49 (13.5)
1000 to <2500 kJ/d	167 (26.7)	82 (22.5)
≥2500 kJ/d	66 (10.6)	42 (11.5)
Age when solids introduced		
≤4 months	140 (22.4)	70 (19.2)
5 months	230 (36.8)	150 (41.2)
6 months	241 (38.6)	134 (36.8)
≥7 months	14 (2.2)	10 (2.8)
Current iron supplement ^l^	4 (0.6)	3 (0.8)

Abbreviations: body mass index, BMI; NZ, New Zealand. ^a^ “Iron status data” defined as all of the following indicators: haemoglobin, plasma ferritin, soluble transferrin receptor (sTfR), C-reactive protein (CRP), and α1-acid glycoprotein (AGP). ^b^ *n* = 1 missing data (participant did not specify sex). ^c^ Participants who reported two or more ethnic groups were assigned to a single group using a prioritisation system [22], with the following order of priority (from highest to lowest): Māori, Pacific, Asian, Others, NZ, and other European. ^d^ Term is defined as 37 weeks gestation or older. ^e^ *n* = 16 missing data in whole sample and *n* = 8 missing data in those with iron status data. BMI z-score was calculated using WHO BMI-for-age child growth standards [25,26]. ^f^ *n* = 2 missing data in whole sample and *n* = 1 missing data in those with iron status data. ^g^ Not employed or is currently on parental leave. ^h^ *n* = 29 missing data in whole sample and *n* = 12 missing data in those with iron status data. Overweight or obese defined as BMI ≥25 kg/m^2^ [37]. ^i^ Formal care is defined as early childhood education centre or home-based care. ^j^ Determined using the New Zealand Index of Deprivation 2018 [23]. ^k^ Infant formula intake is based on mean kilojoule intake per day using data from 24 h diet recalls. ^l^ Iron from iron supplements ranged from 0.084 mg/day to 11.4 mg/day.

**Table 3:** Measures of iron status and inflammation.

	Sample with Iron Status Data (*n* = 364)	Weighted Estimate of Mean or Percentage as Specified (95% CI) ^a^
Haemoglobin (g/L), mean (SD)	115 (9)	115 (114 to 116)
Plasma ferritin ^b^ (μg/L), geometric mean (95% CI)	23.2 (21.4 to 25.1)	22.8 (21.0 to 24.8)
Soluble transferrin receptor ^b^ (mg/L), mean (SD)	4.3 (1.3)	4.3 (4.2 to 4.4)
Body iron (mg/kg), mean (SD)	3.1 (3.2)	3.0 (2.7 to 3.4)
C-reactive protein, % (*n*) ^c^		
≤5 mg/L	95.3 (347)	95.6 (93.0 to 97.3)
>5 mg/L	4.7 (17)	4.4 (2.7 to 7.0)
α1-acid glycoprotein, % (*n*) ^c^		
≤1 g/L	89.0 (324)	89.2 (85.5 to 92.1)
>1 g/L	11.0 (40)	10.8 (7.9 to 14.5)
Iron status categories, % (*n*)		
Iron-sufficient ^d^	77.8 (283)	77.1 (72.3 to 81.3)
Iron-depleted ^e^	9.1 (33)	8.9 (6.3 to 12.3)
Early “functional” iron deficiency ^f^	10.4 (38)	11.1 (8.1 to 15.1)
Iron deficiency anaemia ^g^	2.8 (10)	2.9 (1.5 to 5.5)

^a^ Estimates are weighted for ethnicity and socioeconomic deprivation level to more closely represent the New Zealand population [36]. ^b^ BRINDA (Biomarkers Reflecting Inflammation and Nutritional Determinants of Anaemia) method used to adjust for inflammation [30,31]. ^c^ Cutoff values obtained from Thurnham et al. [38]. ^d^ Defined as plasma ferritin ≥ 15 μg/L in the absence of early “functional” iron deficiency, iron deficiency anaemia, or iron depletion. ^e^ Defined as plasma ferritin < 15 μg/L in the absence of early “functional” iron deficiency and iron deficiency anaemia. ^f^ Defined as body iron < 0 mg/kg and haemoglobin ≥ 105 g/L. ^g^ Defined as body iron < 0 mg/kg and haemoglobin < 105 g/L.

**Table 4:** Baby food pouch use ^a^ at around six months of age and current ^b^ iron status (*n* = 364).

		Non-Pouch User	Non-Frequent Pouch User	Frequent Pouch User
*n*		189	113	62
Current plasma ferritin concentration				
	Geometric mean (95% CI), μg/L	21.0 (18.6, 23.7)	26.3 (23.1, 29.9)	24.9 (20.7, 30.1)
	Percent mean difference (95% CI), %	Reference	**25.2 (4.5, 50.0)**	15.2 (−3.5, 35.6)
	Adjusted ^c^ percent mean difference (95% CI), %	Reference	15.8 (−3.0, 38.1)	5.2 (−15.8, 31.3)
Current body iron				
	Mean (SD), mg/kg	2.63 (3.47)	3.67 (2.77)	3.65 (2.87)
	Mean difference (95% CI), mg/kg	Reference	**1.04 (0.30, 1.78)**	**0.74 (0.01, 1.47)**
	Adjusted ^c^ mean difference (95% CI), mg/kg	Reference	**0.75 (0.02, 1.48)**	0.58 (−0.33, 1.50)
Current iron sufficiency ^d^				
	*n* (%)	134 (70.9)	97 (85.8)	52 (83.9)
	Odds ratio (95% CI)	Reference	**2.49 (1.35, 4.60)**	**2.13 (1.01, 4.50)**
	Adjusted ^c^ odds ratio (95% CI)	Reference	**2.20 (1.15, 4.21)**	1.50 (0.67, 3.39)

Bold text indicates statistically significant difference. ^a^ Pouch use at around 6 months defined as follows: frequent pouch user (given food from a pouch at least five times per week); non-frequent pouch user (given food from a pouch at least once but less than five times per week); non-pouch user (not given food from pouch at around 6 months of age or ever). ^b^ Mean (SD) age at time of iron assessment was 8.3 (0.8) months, ranging from 6.9 to 10.1 months. ^c^ Adjusted for infant age, sex, socioeconomic deprivation, current infant formula intake category, and current iron supplement use. ^d^ Iron sufficiency was defined as follows: plasma ferritin ≥ 15 μg/L, in the absence of iron depletion, early “functional” iron deficiency, and iron deficiency anaemia.

**Table 5:** Baby-led weaning and traditional spoon-feeding ^a^ at around six months of age and current ^b^ iron status (*n* = 364).

		Traditional Spoon-Feeding	Partial Baby-Led Weaning	Full Baby-Led Weaning
*n*		269	42	53
Current plasma ferritin concentration				
	Geometric mean (95% CI), μg/L	23.9 (21.8, 26.1)	20.3 (15.7, 26.3)	22.2 (17.3, 28.5)
	Percent mean difference (95% CI), %	Reference	−15.0 (−34.1, 9.6)	−7.0 (−26.1, 17.0)
	Adjusted ^c^ percent mean difference (95% CI), %	Reference	−14.0 (−32.9, 10.3)	3.5 (−17.6, 30.0)
Current body iron				
	Mean (SD), mg/kg	3.23 (3.07)	2.84 (3.22)	2.84 (3.84)
	Mean difference (95% CI), mg/kg	Reference	−0.39 (−1.43, 0.66)	−0.39 (−1.34, 0.56)
	Adjusted ^c^ mean difference (95% CI), mg/kg	Reference	−0.34 (−1.37, 0.69)	0.02 (−0.92, 0.97)
Current iron sufficiency ^d^				
	*n* (%)	217 (80.7)	28 (66.7)	38 (71.7)
	Odds ratio (95% CI)	Reference	**0.48 (0.24, 0.97)**	0.61 (0.31, 1.19)
	Adjusted ^c^ odds ratio (95% CI)	Reference	0.52 (0.24, 1.13)	0.91 (0.45, 1.87)

Bold text indicates statistically significant difference. ^a^ Feeding approach at around 6 months of age defined as follows: traditional spoon-feeding [always or mostly spoon-fed by adult; four; *n* = 269]; partial baby-led weaning (about half spoon-fed by an adult and half baby feeding themselves); full baby-led weaning (always or mostly baby feeding themselves). ^b^ Mean (SD) age at time of iron assessment was 8.3 (0.8) months, ranging from 6.9 to 10.1 months. ^c^ Adjusted for infant age, sex, socioeconomic deprivation, current infant formula intake category, and current iron supplement use. ^d^ Iron sufficiency was defined as follows: plasma ferritin ≥ 15 μg/L, in the absence of iron depletion, early “functional” iron deficiency, and iron deficiency anaemia.

**Table 6:** Other early feeding practices at around six months of age and current ^a^ iron status (*n* = 364).

Feeding Practices					
Exclusively Breastfeeding up to around 6 Months ^b^		No		Yes	
*n*		223		141	
Current plasma ferritin concentration					
	Geometric mean (95% CI), μg/L	24.1 (21.8, 26.6)		21.8 (19.0, 24.9)	
	Percent mean difference (95% CI), %	Reference		−9.6 (−23.3, 6.6)	
	Adjusted ^c^ percent mean difference (95% CI), %	Reference		9.2 (−8.8, 30.9)	
Current body iron					
	Mean (SD), mg/kg	3.34 (3.10)		2.80 (3.34)	
	Mean difference (95% CI), mg/kg	Reference		−0.54 (−1.22, 0.14)	
	Adjusted ^c^ mean difference (95% CI), mg/kg	Reference		0.13 (−0.62, 0.88)	
Current iron sufficiency ^d^					
	*n* (%)	179 (80.3)		104 (73.8)	
	Odds ratio (95% CI)	Reference		0.69 (0.42, 1.14)	
	Adjusted ^c^ odds ratio (95% CI)	Reference		1.41 (0.79, 2.51)	
Age when solids introduced		≤4 months	5 months	6 months	≥7 months
*n*		70	150	134	10
Current plasma ferritin concentration					
Geometric mean (95% CI), μg/L		24.4 (20.5, 29.0)	25.5 (22.5, 28.9)	21.1 (18.5, 24.1)	13.4 (6.7, 26.9)
Percent mean difference (95% CI), %		15.6 (−7.5, 44.6)	**20.7 (0.8, 44.6)**	Reference	−36.4 (−61.3, 4.6)
Adjusted ^c^ percent mean difference (95% CI), %		5.7 (−15.4, 32.0)	10.8 (−7.0, 32.0)	Reference	**−41.8 (−64.6, −3.8)**
Current body iron					
Mean (SD), mg/kg		3.36 (3.09)	3.48 (3.12)	2.79 (2.17)	0.80 (4.50)
Mean difference (95% CI), mg/kg		0.57 (−0.35, 1.49)	0.68 (−0.06, 1.43)	Reference	−2.00 (−4.04, 0.05)
Adjusted ^c^ mean difference (95% CI), mg/kg		0.25 (−0.67, 1.17)	0.35 (−0.37, 1.08)	Reference	**−2.19 (−4.20, −0.18)**
Current iron sufficiency ^d^					
*n* (%)		57 (81.4)	121 (80.7)	100 (74.6)	5 (50.0)
Odds ratio (95% CI)		1.49 (0.73, 3.05)	1.42 (0.81, 2.49)	Reference	0.34 (0.09, 1.25)
Adjusted ^c^ odds ratio (95% CI)		1.13 (0.52, 2.46)	1.17 (0.65, 2.14)	Reference	**0.21 (0.05, 0.96)**
Red meat consumption ^e^		Never	Less than weekly	Weekly	Daily
*n*		150	23	167	24
Current plasma ferritin concentration					
	Geometric mean (95% CI), μg/L	22.5 (19.8, 25.6)	25.7 (20.8, 31.9)	23.0 (20.4, 26.1)	26.2 (19.5, 35.1)
	Percent mean difference (95% CI), %	Reference	14.3 (−18.9, 61.2)	2.3 (−13.9, 21.6)	16.3 (−17.0, 62.9)
	Adjusted ^c^ percent mean difference (95% CI), %	Reference	12.3 (−19.4, 56.4)	1.3 (−14.2, 19.5)	24.1 (−10.3, 71.8)
Current body iron					
	Mean (SD), mg/kg	2.93 (3.34)	3.88 (2.34)	3.11 (3.24)	3.81 (2.69)
	Mean difference (95% CI), mg/kg	Reference	0.96 (−0.45, 2.37)	0.18 (−0.53, 0.89)	0.88 (−0.50, 2.27)
	Adjusted ^c^ mean difference (95% CI), mg/kg	Reference	0.93 (−0.43, 2.30)	0.14 (−0.54, 0.83)	1.15 (−0.19, 2.49)
Current iron sufficiency ^d^					
	*n* (%)	115 (76.7)	21 (91.3)	127 (76.1)	20 (83.3)
	Odds ratio (95% CI)	Reference	3.20 (0.71, 14.31)	0.97 (0.58, 1.62)	1.52 (0.49, 4.75)
	Adjusted ^c^ odds ratio (95% CI)	Reference	3.40 (0.71, 16.36)	0.96 (0.55, 1.68)	1.73 (0.51, 5.84)
Iron-fortified rice cereal consumption ^e^		Never	Less than weekly	Weekly	Daily
*n*		190	10	72	92
Current plasma ferritin concentration					
	Geometric mean (95% CI), μg/L	23.4 (20.9, 26.2)	24.3 (12.3, 47.9)	24.1 (19.9, 29.3)	21.9 (18.9, 25.3)
	Percent mean difference (95% CI), %	Reference	3.6 (−37.1, 70.4)	3.0 (−16.7, 27.4)	−6.7 (−13.2, 13.4)
	Adjusted ^c^ percent mean difference (95% CI), %	Reference	−0.2 (−39.5, 64.6)	−0.5 (−18.8, 22.0)	−13.3 (−28.5, 5.1)
Current body iron					
	Mean (SD), mg/kg	3.16 (3.31)	3.23 (3.79)	3.38 (3.09)	2.86 (3.02)
	Mean difference (95% CI), mg/kg	Reference	0.08 (−1.97, 2.13)	0.23 (−0.65, 1.10)	−0.30 (−1.10, 0.51)
	Adjusted ^c^ mean difference (95% CI), mg/kg	Reference	−0.03 (−2.09, 2.04)	0.09 (−0.75, 0.93)	−0.58 (−1.37, 0.22)
Current iron sufficiency ^d^					
	*n* (%)	143 (75.3)	8 (80.0)	58 (80.6)	74 (80.4)
	Odds ratio (95% CI)	Reference	1.31 (0.27, 6.41)	1.36 (0.70, 2.66)	1.35 (0.73, 2.49)
	Adjusted ^c^ odds ratio (95% CI)	Reference	1.14 (0.21, 6.23)	1.25 (0.55, 2.56)	1.08 (0.55, 2.10)

Bold text indicates statistically significant difference. ^a^ Mean (SD) age at time of iron assessment was 8.3 (0.8) months, ranging from 6.9 to 10.1 months. ^b^ Exclusively breastfeeding up to around 6 months defined as follows: no (exclusively breastfed up to <5 months); yes (exclusively breastfed up to ≥5 months). ^c^ Adjusted for infant age, sex, socioeconomic deprivation, current infant formula intake category, and current iron supplement use. ^d^ Iron sufficiency was defined as follows: plasma ferritin ≥ 15 μg/L, in the absence of iron depletion, early “functional” iron deficiency, and iron deficiency anaemia. ^e^ Consumption frequency defined as follows: never; less than weekly (1–3 times a month); weekly (1–6 times a week); daily (1 or more times a day).

## Data Availability

The data used in the present study are not publicly available due to ethical restrictions related to the consent provided by participants. An ethically compliant dataset may be made available by the corresponding author upon reasonable request and upon approval by the Health and Disability Ethics Committees New Zealand.

## Supplementary Materials

The following supporting information can be downloaded at: https://www.mdpi.com/article/10.3390/nu16101494/s1, Table S1: Body iron and iron sufficiency by demographic and current feeding characteristics (*n* = 364).
