# Supplementary material for: Baby Food Pouches, Baby-Led Weaning, and Iron Status in New Zealand Infants: An Observational Study
Source: Nutrients. 2024 May 15;16(10):1494. doi: 10.3390/nu16101494 (PMC11124519; doi:10.3390/nu16101494)
Supplement: Supplementary file 1 [file nutrients-16-01494-s001.zip › nutrients-3009481-supplementary.pdf]

Supplementary Materials

Supplementary Table S1. Body iron and iron sufficiency by demographic and current feeding characteristics (n=364).

	n	Body iron, mg/kg		Iron sufficiency[a]	
		Mean (SD)	p-value[b]	n (%)	p-value[b]
**Infant characteristics**					
Infant age			**0.004**		**0.016**
7-7.9 months	143	3.8 (3.2)		119 (83.2)	
8-8.9 months	139	2.6 (3.3)		97 (69.8)	
9-10.0 months	82	2.9 (2.9)		67 (81.7)	
Infant sex[c]			**0.002**		**0.024**
Female	161	3.7 (3.2)		134 (83.2)	
Male	202	2.7 (3.1)		148 (73.3)	
Infant ethnicity[d]			0.267		0.121
Māori	62	3.5 (3.1)		51 (82.3)	
Pacific	14	3.9 (2.0)		13 (92.9)	
Asian	39	2.6 (3.7)		31 (79.5)	
Others	10	1.5 (3.0)		5 (50.0)	
NZ and other European	239	3.1 (3.2)		183 (76.6)	
Infant born at term[e]			**0.001**		**<0.001**
Yes	340	3.3 (3.0)		272 (80.0)	
No	24	1.0 (5.1)		11 (45.8)	
Infant BMI category[f]			0.077		**0.033**
At risk of overweight (BMI z-score >1)	84	2.6 (3.3)		58 (69.1)	
Not overweight (BMI z score ≤1)	272	3.3 (3.2)		218 (80.2)	
**Respondent characteristics**					
Respondent age[g]			0.331		0.319

<25 years	21	4.1 (2.0)		19 (90.5)	
25-35 years	211	3.1 (3.4)		161 (76.3)	
≥35 years	131	3.0 (3.0)		103 (78.6)	
Highest level of education completed			0.461		0.930
School (primary or secondary)	49	2.7 (3.7)		38 (77.6)	
Polytechnic or similar tertiary institution	71	3.4 (3.4)		54 (76.1)	
University	243	3.1 (3.0)		190 (78.2)	
Maternal parity[g]			0.450		0.363
Primiparous	178	3.3 (3.2)		142 (79.8)	
Multiparous	186	3.0 (3.2)		141 (75.8)	
Current employment status			0.700		0.066
Employed full time	45	3.0 (2.6)		39 (86.7)	
Employed part time	84	3.4 (3.0)		70 (83.3)	
Other[h]	235	3.1 (3.4)		174 (74.0)	
Respondent weight status[i]			0.054		0.425
Overweight or obese	217	2.8 (3.3)		164 (75.6)	
Normal weight	135	3.5 (3.0)		107 (79.3)	
Childcare use			0.711		**0.029**
Formal care[j]	66	3.3 (2.5)		58 (87.9)	
No formal care	298	3.1 (3.3)		225 (75.5)	
**Household factors**					
Number of children living in household			0.554		0.427
One	165	3.3 (3.2)		133 (80.6)	
Two	124	3.0 (3.3)		92 (74.2)	
Three or more	74	3.0 (3.1)		57 (77.0)	

Number of adults living in household			0.597		0.532
One	16	3.7 (2.8)		14 (87.5)	
Two	311	3.1 (3.3)		239 (76.9)	
Three or more	37	2.7 (3.0)		30 (81.1)	
Household deprivation decile[k]			0.415		0.620
1-3 (Low)	111	3.1 (3.1)		85 (76.6)	
4-7	165	3.3 (3.2)		132 (80.0)	
8-10 (High)	88	2.8 (3.3)		66 (75.0)	
**Current infant feeding characteristics**					
Ever breastfed			**0.037**		0.104
Yes	355	3.1 (3.2)		274 (77.2)	
No	9	5.3 (2.3)		9 (100)	
Currently breastfed			**<0.001**		**<0.001**
Yes	249	2.6 (3.5)		177 (71.1)	
No	115	4.2 (2.0)		106 (92.2)	
Current infant formula intake[l]			**<0.001**		**<0.001**
No formula	191	2.4 (3.7)		127 (66.5)	
<1000 kJ/d	49	4.0 (3.0)		41 (83.7)	
1000 to <2500 kJ/d	82	3.8 (1.9)		78 (95.1)	
≥2500 kJ/d	42	4.1 (2.1)		37 (88.1)	
Current iron supplement[m]			0.847		0.643
Yes	3	3.5 (4.9)		2 (66.7)	
No	361	3.1 (3.2)		281 (77.8)	

Abbreviations: Body mass index, BMI; NZ, New Zealand.

[a] Iron sufficiency was defined as: Plasma ferritin ≥15 μg/L, in the absence of iron depletion, early “functional” iron deficiency, and iron deficiency anaemia.

[b] Determined using one-way ANOVA F test for body iron and chi-squared test for iron sufficiency.

[c] n=1 missing data (participant did not specify sex).

[d] Participants who reported two or more ethnic groups were assigned to a single group using a prioritisation system [22], with the following order of priority (from highest to lowest): Māori, Pacific, Asian, Others, NZ and other European.

[e] Term is defined as 37 weeks gestation or older.

[f] n=16 missing data; BMI z-score was calculated using WHO BMI-for-age child growth standards [25,26].

[g] n=1 missing data.

[h] Not employed, or is currently on parental leave.

[i] n=29 missing data; Overweight or obese defined as BMI ≥25 kg/m^2 [37].

[j] Formal care is defined as early childhood education centre or home-based care.

[k] Determined using the New Zealand Index of Deprivation 2018 [23].

[l] Infant formula intake is based on mean kilojoule intake per day using data from the 24-hour diet recalls.

[m] Iron from iron supplements ranged from 0.084 mg/day to 11.4 mg/day.
